# Precompetitive anxiety and self-confidence during the 2023 Finnish Padel championship in high level men’s players

**DOI:** 10.3389/fpsyg.2023.1301623

**Published:** 2023-12-18

**Authors:** Rafael Conde-Ripoll, Adrián Escudero-Tena, Vicente Javier Suárez-Clemente, Álvaro Bustamante-Sánchez

**Affiliations:** Faculty of Sport Sciences, Universidad Europea de Madrid, Madrid, Spain

**Keywords:** psychophysiology, confidence, anxiety, competition, racquet sports, Padel, CSAI-2R, STAI-S

## Abstract

The aim of the current study was to assess precompetitive anxiety and self-confidence in high level men’s padel players from Finland. Twenty eight men’s padel players from the highest category participated in the research (87.5% of the target population). The CSAI-2R (Competitive State Anxiety Inventory-2 Revised) and STAI-S (State–Trait Anxiety Inventory – State) questionnaires were used and descriptive and inferential analyzes were performed, including the Kruskal Wallis’s H and Mann–Whitney’s U tests. The results show that seeded players presented lower levels of cognitive anxiety (η^2^ = 0.111). Moreover, losers of first round presented more state anxiety than winners (η^2^ = 0.302). Before the first match, state anxiety was higher than prior to the second match (η^2^ = 0.148). Furthermore, lower ranked players of first round, compared to second, presented more state anxiety and somatic anxiety (η^2^ = 0.487 and η^2^ = 0.277, respectively). However, according to the results obtained, self-confidence was not affected by any of the variables analyzed (ranking, seed, result or round). These findings may be of great interest to players, coaches and sports psychologists, as they allow an enhanced comprehension of the player’s level of anxiety and self-confidence.

## 1 Introduction

Padel is a doubles racquet sport played on a court measuring 20 × 10 meters, divided by a central net, and enclosed by glass or fencing that is four o three meters in height along the court’s sides and bottom, against which the ball can be hit during play (PadelFip: Federación Internacional de Pádel, 2023). Padel has experienced remarkable growth in recent decades, as indicated by Courel Ibáñez et al. (2017). Its popularity is due to the simplicity of its rules and its adaptability in terms of physical and technical-tactical demands, allowing individuals of varying ages and skill levels to take part in the sport (Coruel-Ibáñez et al., 2018; García-Benítez et al., 2018). Presently, this sport boasts a presence in over 70 countries, resulting in a substantial surge in facilities, commercial agreements such as sponsorships and employment contracts, as well as sports licenses in recent years (Muñoz et al., 2016; PadelFip: Federación Internacional de Pádel, 2023). Within this context, the quantity of scientific studies pertaining to padel has seen an uptick in recent years (García-Giménez et al., 2022; Martín-Miguel et al., 2023; Sánchez-Alcaraz et al., 2023). Among the various topics under investigation, the analysis of performance stands out as the most frequently studied aspect. Research carried out on performance analysis seeks to determine the differences that exist between players based on their level of play (Muñoz et al., 2017a,b), gender (Escudero-Tena et al., 2021a, 2022b) or result (Escudero-Tena et al., 2021b, 2022a).

Mental toughness is essential for attaining success in sports (Crust and Keegan, 2010) and sustaining and enhancing performance in competition (Gucciardi et al., 2015). Moments right before the actual competition begins, players tend to experience anxiety (Dosil, 2004). Such anxiety, commonly recognized as precompetitive anxiety (Cox, 2012), has been extensively studied in the recent past (Cuesta-Vargas and Vertedor Corpas, 2016; Correia and Rosado, 2019; Pineda-Espejel et al., 2021; Ren et al., 2022). This mental factor is widely acknowledged to have a profound impact on performance in sports competition, in addition to self-confidence (León-Prados et al., 2014; Pineda-Espejel et al., 2019). Sport-related research has demonstrated that athletes displaying greater amounts of anxiety typically perform worse in competitions compared to those with less anxiety (León-Prados et al., 2011; López-Torrers et al., 2011; Ngo et al., 2017; Sánchez et al., 2017). However, the classic assertion that anxiety invariably has a detrimental impact on sports performance has come under scrutiny. Elite athletes often perceive anxiety symptoms as performance-enhancing, while those with less expertise tend to view anxiety as hindering their performance. In fact, experiencing competitive anxiety can be advantageous for performance, as long as the athlete maintains control (Jones and Hanton, 2001; Demarie et al., 2013). In any case, it may seem important to manage and control that precompetitive anxiety to achieve optimal sport performance. Furthermore, the player’s self-confidence, that is, the player’s conviction in their potential for successful competition (Robazza and Bortoli, 2007), is positively correlated with sporting success (Santos-Rosa et al., 2007; Díaz et al., 2008; Martínez-Romero et al., 2016; Zurita-Ortega et al., 2017). Nevertheless, an excessive level of self-confidence is capable of decreasing the ideal performance level (Weinberg and Gould, 2010). The correlation between an athlete’s self-confidence and their competitive performance stands out as one of the most relevant aspects in the realm of sports performance (Vodičar et al., 2012).

Several factors, such as the sex and type of sport, can potentially influence precompetitive anxiety (Martens et al., 1990). It is worth noting that, in racquet sports, players have to constantly make decisions in short periods of time (Castillo-Rodríguez et al., 2014) and coping with high-pressure situations is intrinsically linked to performance (González-Díaz et al., 2012; Knight et al., 2016; Martínez-Gallego et al., 2022). In general, in individual sports, compared to team sports where responsibility is shared among all team members, athletes present an increased amount of precompetitive anxiety (Koronas et al., 2020). However, according to Rodríguez-Cayetano et al. (2022), when comparing tennis (singles format) to padel (doubles format), the results are not like this in every case, differing depending on the sex. These authors found that women’s tennis players, compared to padel players of the same sex, have a significantly higher level of cognitive anxiety and a significantly lower level of self-confidence, but no differences were found in the somatic anxiety. In addition, the same authors indicate that men’s padel players, compared to tennis players of the same sex, present significantly higher level of somatic anxiety but no differences have been found in cognitive anxiety and self-confidence. It is also worth highlighting that senior padel players, compared to junior padel players, show significantly higher levels of cognitive and somatic anxiety and a significantly lower level of self-confidence.

After analyzing the scientific literature, as indicated above, in padel there has been an increase in scientific publications in recent times (García-Giménez et al., 2022; Martín-Miguel et al., 2023). However, mental preparation and performance have been scarcely studied, with only a small number of studies that have analyzed the level of pre-competitive anxiety (Almendros-Pacheco et al., 2022; Castillo-Rodriguez et al., 2022; Rodríguez-Cayetano et al., 2022). Gaining a better understanding of the player’s level of anxiety and self-confidence can be of great interest for players, coaches and sports psychologists. The former can adapt their lifestyle and game style, while the latter can tailor feedback and training sessions accordingly. The aim of the present investigation was to analyze the pre-competitive anxiety and self-confidence during the competition in high-level padel players from Finland according to the ranking, the seed, the result and the round. Therefore, the following hypotheses were put forward: (1) Finnish padel players, compared to players from other nations, will display lower levels of self-confidence and higher levels of anxiety, (2) seeded players, compared to non-seeded, will show less anxiety and more self-confidence; (3) higher ranked players, compared to lower ranked, will show more self-confidence and less anxiety; (4) winners of the matches, compared to losers, will have a higher level of self-confidence and a lower level of anxiety; (5) in first round, lower ranked players and losers will present more anxiety and less self-confidence than higher ranked players and winners, respectively; (6) first round matches, compared to second, will bring more anxiety for the players and the players will have less self-confidence; (7) as rounds go by, players with lower ranking will present more anxiety and less self-confidence.

## 2 Materials and methods

### 2.1 Participants

A total of 28 men’s high level padel players from Finland (87.5% of the target population) voluntarily participated in the present study, which took place during the main draw of the 2023 Finnish Padel Championship. The obtained points of this competition counted for the ranking of the Finnish Federation. 23 (71.9%), 12 (75.0%), 8 (100%), and 4 (100%) players completed the questionnaires in round of 16 (first round), quarter finals, semifinals and final, respectively. Participants completed the questionnaires in English, as it is the only language that both researchers and athletes are fluent in. All participants were ranked top 103 in Finland. At the time of the measurements, none of the athletes had physical injuries, nor were they using any medication. Furthermore, none of the participants had any hindrance to their involvement in the study. The study was in accordance with the Helsinki Declaration (World Medical Association, 2013). Participants were treated ethically under the American Psychological Association code of ethics regarding consent, anonymity and responses. Previously, the current investigation had been approved by the Ethics Committee of the European University of Madrid with the code CIPI/22.303. To obtain permission to administer the questionnaires to the players before the competition, the researchers first contacted the Finnish Padel Federation and the championship organizer. So as to respect the principles of voluntariness and confidentiality, each player was required to sign an informed consent form that clearly explained the objectives of the research and their voluntary participation in it.

### 2.2 Instruments

#### 2.2.1 Competitive anxiety

CSAI-2R was used to measure precompetitive anxiety and self-confidence of padel players. This inventory was originally developed by Martens et al. (1990) to measure the intensity of cognitive and somatic responses and self-confidence before training and before competition. It was adapted and validated years later by Cox (2012). The CSAI-2R consists of 17 items (using a 4-point Likert scale). These items make up a total of three subscales: cognitive anxiety, somatic anxiety and self-confidence. Higher values represented higher cognitive anxiety, somatic anxiety, and self-confidence levels. In the analysis of the instrument, Cronbach’s alpha coefficients were obtained, showing reliability scores of 0.72 for cognitive anxiety, 0.66 for somatic anxiety, and 0.85 for self-confidence, all meeting acceptable standards (Cortina, 1993; Nunnally and Bernstein, 1994; DeVellis, 2003; Vaske, 2008).

#### 2.2.2 State anxiety

STAI-S was used to measure state anxiety. This inventory was developed by Spielberger et al. (1970). This questionnaire has been used in previous research in racquet sports (García-Gonzálvez et al., 2022; Villafaina et al., 2022).

#### 2.2.3 Procedure

The questionnaires were administered to the players between 30 and 45 min prior to the start of each match, following similar criteria to that used by Andrade Fernández et al. (2007), who administered their questionnaires between 15 and 45 min before the start of the competition. All questionnaires were completed in a quiet room with a controlled temperature of 20°C. Participants were not allowed to speak during the assessments.

### 2.3 Statistical analysis

All data were analyzed using the statistical package SPSS for Macintosh v.25.0 (SPSS Inc., Chicago, IL, United States). A Kolgomorov-Smirnov test was used to test the normality of the distribution of the data and it indicated that it is non-parametric. Then, a descriptive analysis was performed to obtain information on the number of times the categories of each study variable occurred (mean, standard deviation, median, maximum, and minimum).

Next, inferential analyzes were carried out to analyze the differences between the anxiety factors and the self-confidence with the independent variables result (winner/loser), ranking (higher/lower), seed (seeded/non-seeded), round (first round, quarter-final, semi-final, final). Mann–Whitney’s U and Kruskal Wallis’s H tests were used. A *p* value of less than 0.05 was considered to be statistically significant.

## 3 Results

Table 1 shows the descriptive analysis of the psychological variables regarding precompetitive anxiety and self-confidence.

**Table 1:** Descriptive analysis of the precompetitive anxiety and self-confidence.

Variable	Mean	SD	Median	Max.	Min.
CA	1.37	0.39	1.20	2.40	1.00
SA	1.59	0.37	1.57	2.71	1.00
SC	3.21	0.59	3.20	4.00	1.40
STA	7.23	3.41	6.00	16.00	2.00

CA, cognitive anxiety; SA, somatic anxiety; SC, self-confidence; STA, state anxiety; SD, standard deviation; Max., maximum; Min., minimum.

Table 2 shows the level of precompetitive anxiety and self-confidence as a function of the players being seeded or not in the draw. Significant differences (*p* = 0.018; η^2^ = 0.111) were found in cognitive anxiety.

**Table 2:** Precompetitive anxiety and self-confidence between seeded and non-seeded players.

	Seeded players (*n* = 25)	Non seeded players (*n* = 22)		
Variable	Median (IQR)	Median (IQR)	*p*	η^2^
CA	1.20 (0.10)	1.50 (0.85)	0.018*	0.111
SA	1.57 (0.57)	1.71 (0.36)	0.337	0.019
SC	3.40 (0.80)	3.20 (0.60)	0.091	0.060
STA	6.00 (2.50)	7.00 (6.00)	0.095	0.058

CA, cognitive anxiety; SA, somatic anxiety; SC, self-confidence; STA, state anxiety; n, number; IQR, interquartile range; *p*, value of *p*; *p < 0.05; η^2^, eta squared.

Table 3 shows the level of precompetitive anxiety and self-confidence as a function of the ranking (higher/lower) of the pairs confronted in each match. No significant differences were found.

**Table 3:** Precompetitive anxiety and self-confidence according to the ranking.

	Higher ranked (*n* = 22)	Lower ranked (*n* = 25)		
Variable	Median (IQR)	Median (IQR)	*p*	η^2^
CA	1.20 (0.25)	1.20 (0.70)	0.938	0.000
SA	1.64 (0.71)	1.57 (0.29)	0.650	0.004
SC	3.40 (0.80)	3.20 (0.70)	0.485	0.010
STA	6.00 (2.25)	7.00 (6.00)	0.174	0.038

CA, cognitive anxiety; SA, somatic anxiety; SC, self-confidence; STA, state anxiety; n, number; IQR, interquartile range; *p*, value of *p*; *p < 0.05; η^2^, eta squared.

Table 4 shows the level of precompetitive anxiety and self-confidence as a function of the result (winner/loser) of each match. No significant differences were found.

**Table 4:** Precompetitive anxiety and self-confidence according to the result.

	Match winner (*n* = 22)	Match loser (*n* = 25)		
Variable	Median (IQR)	Median (IQR)	*p*	η^2^
CA	1.20 (0.30)	1.20 (0.70)	0.588	0.006
SA	1.57 (0.61)	1.71 (0.36)	0.222	0.031
SC	3.40 (0.80)	3.20 (0.90)	0.150	0.043
STA	6.00 (1.25)	8.00 (6.00)	0.054	0.077

CA, cognitive anxiety; SA, somatic anxiety; SC, self-confidence; STA, state anxiety; n, number; IQR, interquartile range; *p*, value of *p*; *p < 0.05; η^2^, eta squared.

Table 5 shows the level of precompetitive anxiety and self-confidence as a function of the result in round of 16 (first round) of the championship. Significant differences (*p* = 0.006; η^2^ = 0.302) were found in state anxiety.

**Table 5:** Precompetitive anxiety and self-confidence according to the result in round of 16.

	Match winner (*n* = 10)	Match loser (*n* = 13)		
Variable	Median (IQR)	Median (IQR)	*p*	η^2^
CA	1.20 (0.60)	1.40 (1.00)	0.313	0.046
SA	1.50 (0.61)	1.71 (0.36)	0.148	0.092
SC	3.10 (1.05)	3.00 (0.60)	0.648	0.009
STA	6.00 (2.25)	11.00 (5.00)	0.006*	0.302

CA, cognitive anxiety; SA, somatic anxiety; SC, self-confidence; STA, state anxiety; n, number; IQR, interquartile range; *p*, value of *p*; *p < 0.05; η^2^, eta squared.

Table 6 shows the level of precompetitive anxiety and self-confidence between the round of 16 (first round) and quarter finals. Significant differences were found in state anxiety (*p* = 0.022; η^2^ = 0.148).

**Table 6:** Precompetitive anxiety and self-confidence between round of 16 and quarter finals.

	R16 (*n* = 23)	QF (*n* = 12)		
Variable	Median (IQR)	Median (IQR)	*p*	η^2^
CA	1.20 (1.00)	1.20 (0.30)	0.290	0.030
SA	1.71 (0.57)	1.43 (0.43)	0.071	0.092
SC	3.00 (0.60)	3.40 (0.70)	0.082	0.085
STA	7.00 (5.00)	5.50 (2.50)	0.022*	0.148

CA, cognitive anxiety; SA, somatic anxiety; SC, self-confidence; STA, state anxiety; R16, round of 16; QF, quarter finals; n, number; IQR, interquartile range; *p*, value of *p*; *p < 0.05; η^2^, eta squared.

When comparing other rounds, significant differences (*p* = 0.020; η^2^ = 0.332) were only found between quarter finals and final in state anxiety.

Table 7 shows the level of precompetitive anxiety and self-confidence as a function of the round for the lower ranked players of each match. Significant differences were found in somatic anxiety (*p* = 0.032; η^2^ = 0.277) and state anxiety (*p* = 0.004; η^2^ = 0.487).

**Table 7:** Precompetitive anxiety and self-confidence as a function of the round in lower ranked players.

	R16 (*n* = 13)	QF (*n* = 6)	SF (*n* = 4)	*F* (*n* = 2)		
Variable	Median (IQR)	Median (IQR)	Median (IQR)	Median (IQR)	*p*	η^2^
CA	1.40 (1.00)	1.20 (0.65)	1.10 (0.35)	1.20 (0.00)	0.438	0.014
SA	1.71 (0.36)	1.43 (0.32)	1.50 (0.46)	1.64 (0.07)	0.032*	0.277
SC	3.00 (0.60)	3.30 (0.50)	3.20 (0.50)	3.60 (0.40)	0.363	0.009
STA	11.00 (5.00)	5.00 (2.00)	5.00 (0.75)	8.50 (0.50)	0.004*	0.487

CA, cognitive anxiety; SA, somatic anxiety; SC, self-confidence; STA, state anxiety; R16, round of 16; QF, quarter finals; SF, semifinals; F, final; n, number; IQR, interquartile range; *p*, value of *p*; *p < 0.05; η^2^, eta squared.

The differences were found between round of 16 (first round) and quarter finals in somatic anxiety (*p* = 0.009; η^2^ = 0.329) and state anxiety (*p* = 0.002; η^2^ = 0.428). Also, there were significant differences between round of 16 (first round) and semifinals in state anxiety (*p* = 0.010; η^2^ = 0.349).

## 4 Discussion

The aim of the present study was to evaluate anxiety and self-confidence prior to sports competition in high level men’s padel players from Finland. As an initial hypothesis it was established that Finnish padel players would have lower self-confidence values and higher anxiety levels. This hypothesis was accepted in part, since Finnish padel players showed lower values of self-confidence compared to Spanish padel players from the same category (Castillo-Rodriguez et al., 2022). However, Finnish padel players also presented lower values of cognitive anxiety, and similar values of somatic anxiety. This could be since padel in Finland is a relatively new sport. Thus, padel players from Spain may have more experience, and cognitive anxiety and self-confidence are directly and positively influenced by experience (Castillo-Rodriguez et al., 2022).

Another hypothesis was that seeded players, compared to non-seeded, would present less anxiety and more self-confidence. This hypothesis was accepted in part, since seeded players showed significantly lower levels of cognitive anxiety. This may be attributed to the fact that by having a higher ranking, seeded players have more competitive experience and technical-tactical abilities, which could contribute to present less cognitive anxiety. In view of the results, it is advisable for non-seeded players to incorporate a psychological skills training program to reduce this elevated level of cognitive anxiety (Mathers and Brodie, 2011; Slack et al., 2015). It is also recommended to include pressure during training through the implementation of psychological demands and consequences that leave a sustained mark on athletes (Stoker et al., 2016; Low et al., 2023). Nevertheless, contrary to our findings, Castillo-Rodriguez et al. (2022) found that the higher the level of the padel players, the higher the values of self-confidence and the lower the values of somatic anxiety. It is important to note that in the cited study, this comparison was made among 3 level of categories and there was no comparison intra-category, as it is the case in our study.

It was also hypothesized that players with higher ranking would display more self-confidence and lower anxiety than players with lower ranking. This hypothesis was not accepted at all. There were no significant differences despite being the values as expected. This could be due to the lack of experience of the participants since, as previously indicated, padel is a new sport in Finland. And it has been shown that self-confidence is affected by the experience (Castillo-Rodriguez et al., 2022).

Another hypothesis was that winners of the match would present higher level of self-confidence and lower level of anxiety compared to losers before matches. This hypothesis was not accepted at all. Despite the differences were as expected, these were not significant. This could be partly explained since the padel players increase their self-confidence during and after the game (Rodríguez-Cayetano et al., 2017).

Additionally, it was hypothesized that in first round, losers would present more pre-competitive anxiety than winners, and less self-confidence. This hypothesis was accepted in part. Significant differences were found in the level of state anxiety. This result could be explained by the fact that a high level of anxiety indicates a decrease in sports performance (Garcia-Mas et al., 2011; Ries et al., 2012).

Furthermore, there was a hypothesis suggesting that first round matches (round of 16), compared to second (quarterfinals), would involve more anxiety and less self-confidence for the players. This hypothesis was accepted in part. Significant differences were found in state anxiety. To the best of our knowledge, there are no other studies in padel regarding this particular topic. Nevertheless, these results are partially in line with the study of Villafaina et al. (2022), in which it was found that elite international junior tennis players had lower state anxiety levels before the second match than before the first match of the same competition. Besides, prior to the first match, compared to the second, the level of cognitive anxiety was also higher in these tennis players. Concordantly, BMX cyclists experienced significant decreases in both somatic and cognitive anxiety levels as they transitioned from the first round to the second round (Mateo et al., 2012). The authors proposed that these findings might be attributed to the repeated exposure to precompetitive pressure, leading to a diminished fear of failure during competition. Consequently, this reduced the anxiety response among the athletes. Furthermore, a prior study demonstrated that athletes’ prior exposure to a particular competitive environment had an impact on their precompetitive anxiety levels (Cerin et al., 2000).

As a final hypothesis, it was established that, as rounds go by, players with lower ranking would present more anxiety and less self-confidence. This hypothesis was accepted in part. Significant differences were found in the level of somatic anxiety and state anxiety. Ranking is directly associated to the level meaning that low ranked players do not usually reach final rounds in tournaments. And a strong consensus exists, affirming an inverse correlation between anxiety levels and sports performance (León-Prados et al., 2011).

This study boasts various strengths. To begin with, it is the first study carried out with Finnish players. Secondly, it is the first study that compares the level of precompetitive anxiety and self-confidence as a function of the ranking of the players, and the round and outcome of the matches. Thirdly, the findings carry significant practical implications for coaches and sport psychologists, particularly considering the differences in rounds, ranking and outcome of the matches. The results must be taken into account to design effective training programs for each individual athlete, as well as for the design of specific training tasks. For example, exercises that simulate real competition situations that imply a higher degree of anxiety and a lower degree of self-confidence should be an important part of training sessions, such as tie-breaks, golden points, conditional rules of the game, score against, etc.

While this study adopts a similar methodology to recent research in the field, it is essential to underscore certain limitations inherent in this investigation. In future studies, researchers are encouraged to examine whether anxiety and self-confidence responses manifest similarly in both sexes. To measure precompetitive anxiety and self-confidence, it would be advisable to use other tools such as pulsometers which measure heart rate variability, and not only questionnaires. Future research should take into account both precompetitive anxiety and self-confidence and post-competitive anxiety and self-confidence, and relate it to sports performance, at elite and amateur level.

## 5 Conclusion

The levels of anxiety and self-confidence before the sports competition have been described in high-level men’s padel players from Finland according to classification, match result and round.

Anxiety was affected by the variables analyzed (ranking, seed, result, and round). The seeded athletes presented lower levels of cognitive anxiety than the unseeded ones, the losers presented more state anxiety than the winners in the first round and, the state anxiety was also higher before the first round (round of 16) than before the second round (quarterfinals). Finally, the lower ranked players in the first round (round of 16), compared to those in the second (quarterfinals), presented a higher degree of state anxiety and somatic anxiety.

On the other hand, self-confidence was not affected by any of the variables analyzed (ranking, seed, result and round).

As a practical application, based on these results, players are encouraged to develop their mental skills to enhance their performance. Thus, padel coaches should consider undergoing psychological training to provide more effective support to their athletes, as well as the latter could benefit significantly from having a sport psychologist within their teams.

## Data availability statement

The raw data supporting the conclusions of this article will be made available by the authors, without undue reservation.

## Ethics statement

The studies involving humans were approved by the Ethics Committee of the European University of Madrid with the code CIPI/22.303. The studies were conducted in accordance with the local legislation and institutional requirements. The participants provided their written informed consent to participate in this study.

## Author contributions

RC-R: Conceptualization, Data curation, Formal analysis, Investigation, Methodology, Project administration, Software, Writing – original – draft, Writing – review – & – editing. AE-T: Software, Supervision, Validation, Writing – review & editing. VS-C: Funding acquisition, Supervision, Validation, Writing – review & editing. ÁB-S: Conceptualization, Funding acquisition, Project administration, Supervision, Validation, Writing – review & editing.
